# Miltefosine and emerging *Leishmania* (*Mundinia*) in Southeast Asia: Molecular insights, therapeutic challenges, and future strategic implementation

**DOI:** 10.1371/journal.pntd.0014555

**Published:** 2026-07-31

**Authors:** Paul Gabriel Escalera Lerona, Anupop Jitmuang, Patsharaporn Techasintana Sarasombath, Methee Chayakulkeeree, Rawadee Kumlert, Padet Siriyasatien, Alistair C. Darby, Kanok Preativatanyou

**Affiliations:** 1 Biomedical Sciences and Biotechnology Program, Faculty of Medicine, Chulalongkorn University, Bangkok, Thailand; 2 Center of Excellence in Vector Biology and Vector‑Borne Disease, Chulalongkorn University, Bangkok, Thailand; 3 Institute of Infection, Veterinary and Ecological Sciences, University of Liverpool, Liverpool, United Kingdom; 4 Division of Infectious Diseases and Tropical Medicine, Department of Medicine, Faculty of Medicine Siriraj Hospital, Mahidol University, Bangkok, Thailand; 5 Siriraj Integrative Center for Neglected Parasitic Diseases, Department of Parasitology, Faculty of Medicine Siriraj Hospital, Mahidol University, Bangkok, Thailand; 6 Department of Disease Control, Ministry of Public Health, Nonthaburi, Thailand; 7 Department of Parasitology, Faculty of Medicine, Chulalongkorn University, Bangkok, Thailand; 8 Health Protection Research Unit in Gastrointestinal Infections, University of Liverpool, Liverpool, United Kingdom; Advanced Centre for Chronic and Rare Diseases, INDIA

## Abstract

Miltefosine, the only oral antileishmanial drug with regulatory approval, has expanded treatment options in several endemic settings but shows variable efficacy across *Leishmania* species, clinical forms, and host immune contexts. In Southeast Asia, where *Leishmania* (*Mundinia*) *martiniquensis* and *L.* (*M.*) *orientalis* are increasingly reported, amphotericin B formulations remain the main treatment despite toxicity, relapse, and implementation constraints. This review evaluates miltefosine’s clinical relevance, mechanisms of action, and resistance pathways, with emphasis on Thailand and neighboring Southeast Asian settings. A Thai compassionate-use case using miltefosine with liposomal amphotericin B for refractory *L. martiniquensis* infection achieved repeated clinical improvement and culture negativity after combination induction, although monotherapy was insufficient to maintain remission in advanced immunosuppression. Evidence from other endemic regions indicates that poor adherence, unregulated access, prolonged subtherapeutic exposure, and inadequate monitoring can reduce treatment durability and favor reduced susceptibility. Mechanistic studies in non-*Mundinia* species identify transport disruption, lipid and sterol remodeling, mitochondrial stress adaptation, redox buffering, and host-parasite effects as resistance-relevant axes, while regional data raise concern for amphotericin B-associated reduced miltefosine susceptibility in *L. martiniquensis*. Wider implementation should therefore be linked to species-resolved diagnostics, baseline susceptibility testing, longitudinal phenotype-genotype surveillance, drug stewardship, and One Health monitoring. Miltefosine should be considered a rational but carefully monitored therapeutic addition for emerging Southeast Asian leishmaniasis.

## Author summary

Leishmaniasis is a neglected infectious disease affecting vulnerable populations worldwide. In Southeast Asia, autochthonous infections caused by *Leishmania* (*Mundinia*) *martiniquensis* and *L.* (*M.*) *orientalis* are increasingly reported, while available treatments remain toxic, costly, or difficult to administer. Miltefosine, the only oral drug approved for leishmaniasis, has been widely used in other endemic regions but remains underused in Southeast Asia. This review examines its mechanisms of action, resistance pathways, and potential role in Thailand. We discuss a compassionate-use case in which miltefosine combined with liposomal amphotericin B achieved repeated clinical improvement and parasite culture negativity in an immunosuppressed patient with limited treatment options, although miltefosine alone did not sustain remission. Incomplete or poorly monitored miltefosine use can reduce treatment durability and favor reduced susceptibility. Strategic implementation, including improved parasite identification, baseline susceptibility testing, genomic surveillance, and One Health vector and reservoir monitoring, could expand treatment options while protecting long-term efficacy in Southeast Asia.

## 1. Introduction

Leishmaniasis encompasses a spectrum of diseases caused by protozoan parasites of the genus *Leishmania*, transmitted primarily through the bite of infected phlebotomine sand flies [1]. Clinical manifestations range from self-limiting cutaneous ulcers to potentially fatal visceral disease involving the liver, spleen, and bone marrow [2]. Globally, over one billion people are estimated to be at risk, with 600,000–1 million new cases of cutaneous leishmaniasis (CL) and 50,000–90,000 new cases of visceral leishmaniasis (VL) reported annually [3]. CL is highly prevalent in the Americas, the Mediterranean basin, the Middle East, and Central Asia, whereas VL occurs predominantly in Brazil, East Africa, and the Indian subcontinent [4].

The geographic distribution and prevalence of *Leishmania* parasites are driven by interacting ecological, environmental, and socioeconomic factors [5,6]. For example, *L. infantum* is endemic in the Mediterranean basin and Latin America, where domestic dogs serve as major reservoir hosts and adapted sand fly vectors sustain transmission [6]. Socioeconomic conditions remain fundamental determinants of disease burden: poverty, malnutrition, inadequate housing, and limited access to healthcare increase vulnerability to infection, worsen clinical outcomes, and compound the stigma and economic exclusion associated with the disease [7]. Immunocompromised populations, particularly people living with HIV (PLWH), are at increased risk of disseminated or diffuse disease, chronic relapse, and reduced treatment efficacy [8]. Poverty-associated barriers to diagnosis, regulated drug access, and treatment completion can create conditions that favor antimicrobial resistance, although this evidence is broader than leishmaniasis alone. In leishmaniasis, irregular treatment, incomplete courses, and unsupervised access contributed to antimonial resistance in the Indian subcontinent, while analogous concerns have been raised for miltefosine because of its high cost, long elimination half-life, and 28-day regimen [9,10]. The documented distribution of a poor-quality generic miltefosine product without detectable active ingredient in Bangladesh further emphasizes the importance of drug-quality assurance in VL elimination programmes [11].

Miltefosine (hexadecylphosphocholine), originally developed as an anticancer agent [12], was repurposed as the first and only oral antileishmanial drug following successful clinical trials against *L. donovani* in India [13]. It received regulatory approval in India in 2002, with subsequent approvals in Germany (2004), Colombia (2005), and the United States (2014), and was added to the WHO Model List of Essential Medicines in 2011 [14,15]. Its oral bioavailability and suitability for outpatient administration offer substantial logistical advantages over parenteral regimens in settings with limited inpatient capacity [15]. Miltefosine is used in selected regional treatment regimens for VL and post-kala-azar dermal leishmaniasis (PKDL), and the WHO specifically recommends its use in combination with liposomal amphotericin B for HIV co-infected VL patients in East Africa and South-East Asia. Clinical efficacy varies across *Leishmania* species, geographic regions, and host immune status, with declining effectiveness, relapse, and gastrointestinal toxicity documented under routine programmatic conditions for *L. donovani* in the Indian subcontinent, although comparable clinical decline has not been directly demonstrated in *Mundinia* species [16–18]. Restricted global distribution, limited production capacity, teratogenic risk, and the emergence of resistance in endemic areas further constrain its utility, although clinical miltefosine resistance has not yet been directly demonstrated in *Mundinia* species [19,20].

In Southeast Asia, leishmaniasis caused by species within the subgenus *Mundinia* presents a distinct epidemiological and therapeutic landscape. Autochthonous infections caused by *L. martiniquensis* and *L. orientalis* have been increasingly reported in Thailand over the past two decades, with clinical presentations spanning VL, CL, diffuse CL, mucocutaneous leishmaniasis, and asymptomatic infections [21–24]. These *Mundinia* species diverge from the *L. donovani* complex in phylogeny, genome structure, and probable vectors [25]. Molecular surveys and experimental infections have implicated *Culicoides* biting midges (Diptera: Ceratopogonidae) as plausible vectors, distinguishing *Mundinia* transmission from classical sand fly-centered models [26–30]. A disproportionate burden falls on PLWH, who account for approximately half of reported Thai VL cases and experience high rates of relapse, disseminated disease, atypical manifestations, and treatment failure despite amphotericin B-based regimens [23,24,31–34]. Miltefosine is not currently included in Thailand’s National List of Essential Medicines [32,35,36], and circulating *Leishmania* populations in the region remain largely naïve to alkylphospholipid exposure [36,37]. The convergence of atypical vector biology, a vulnerable patient population, limited treatment options, and a miltefosine-naïve parasite population defines a therapeutic setting that differs fundamentally from the Indian subcontinent and Latin American contexts in which miltefosine was developed and deployed.

This review evaluates miltefosine as a rational addition to the limited therapeutic options for *Leishmania* (*Mundinia*) infections in Southeast Asia by integrating molecular, clinical, and ecological evidence. Mechanistic data derived primarily from *L. donovani*, *L. infantum*, *L. major*, and other well-studied species are assessed for their therapeutic and resistance implications, with explicit acknowledgment that direct validation in *Mundinia* species remains largely absent. This review includes a detailed clinical account of the first successful compassionate use of miltefosine in Thailand, administered with liposomal amphotericin B for refractory *L. martiniquensis* infection, based on institutional case records and clinical data provided by co-authors involved in the patient’s management. Clinical images from this case were previously published by Jitrukthai and Charatcharoenwitthaya [38]. This review further proposes a framework for evidence-based introduction of miltefosine in Southeast Asia, integrating species-resolved diagnostics, genomic surveillance, and One Health stewardship to preserve therapeutic efficacy while addressing the distinct epidemiological characteristics of this emerging disease setting.

## 2. Methods

### 2.1 Ethics statement

This review article synthesizes publicly available literature. The compassionate-use case discussed in this review was conducted with written informed consent obtained from the patient for the use and publication of clinical and medical information, which was approved by the Faculty of Medicine Siriraj Hospital, Mahidol University. The broader research initiative related to this work was conducted under ethical approval from the Institutional Review Board of the Faculty of Medicine, Chulalongkorn University, Bangkok (IRB No. 0286/67, COA Nos. 0757/2024, 0700/2025). No additional ethical approval was required.

### 2.2  Literature search and evidence synthesis

This narrative review was prepared through a targeted literature search of PubMed, Web of Science, Scopus, Google Scholar, and Crossref for articles relevant to miltefosine use, mechanisms of action, resistance, diagnostics, and implementation in leishmaniasis, with emphasis on Southeast Asia and *Leishmania* (*Mundinia*) species. Searches were conducted for articles published up to April 2026 using combinations of the terms “miltefosine,” “leishmaniasis,” “Leishmania,” “Mundinia,” “Leishmania martiniquensis,” “Leishmania orientalis,” “Leishmania siamensis,” “Thailand,” “Southeast Asia,” “drug resistance,” “miltefosine resistance,” “amphotericin B resistance,” “cross-resistance,” “miltefosine transporter,” “Ros3,” “ABC transporter,” “lipid metabolism,” “sterol biosynthesis,” “mitochondria,” “calcium homeostasis,” “redox stress,” “apoptosis-like cell death,” “immunomodulation,” “diagnostics,” “genomic surveillance,” “parasitomics,” and “One Health.” Additional sources were identified from reference lists of selected articles and WHO guidance documents.

Articles were prioritized when they addressed one or more of the following: clinical use of miltefosine in visceral, cutaneous, mucocutaneous, disseminated, or HIV-associated leishmaniasis; experimental or clinical evidence for miltefosine susceptibility or resistance; mechanisms of drug uptake, efflux, lipid remodeling, mitochondrial toxicity, redox buffering, host immunomodulation, or parasite survival adaptation; autochthonous leishmaniasis in Thailand or Southeast Asia; diagnostic or surveillance approaches relevant to *Mundinia* species; and implementation or stewardship considerations. Experimental evidence was appraised according to evidence type, distinguishing functional validation studies, laboratory-selected resistant lines, clinical isolate observations, omics associations, pharmacological associations, and hypotheses. Mechanistic findings derived from non-*Mundinia* species were interpreted as extrapolative unless direct evidence was available for *L. martiniquensis*, *L. orientalis*, or other *Mundinia* species.

## 3. *Leishmania* (*Mundinia*) and the emerging leishmaniasis landscape in Southeast Asia

### 3.1 Taxonomy and phylogenetic position of *Mundinia*

The genus *Leishmania* has undergone several taxonomic revisions as molecular approaches have refined our understanding of relationships previously inferred from morphology, clinical presentation, and geographic distribution [39]. Multilocus enzyme electrophoresis, ribosomal DNA sequencing, and whole-genome analyses have supported the current classification of the genus into four subgenera: *Leishmania*, *Viannia*, *Sauroleishmania*, and *Mundinia* (formerly known as the “*Leishmania enriettii* complex”) [25,39]. Comparative genomics and phylogenomic analyses generally place *Mundinia* as the earliest-diverging lineage within the genus *Leishmania*. Members of this subgenus possess relatively smaller genomes than many species belonging to the subgenera *Leishmania* and *Viannia* and may exhibit alternative vector associations, including suspected transmission by non-phlebotomine insects in some species [25]. Genomic reduction in *Mundinia* has been associated with gene losses exceeding gains and gene family contractions exceeding expansions at the *Mundinia* node. Reported changes include losses affecting parasite surface architecture, particularly β-amastins and lipophosphoglycan-modifying enzymes, as well as contractions in oxygen-sensing adenylate cyclases and FYVE zinc finger-containing proteins [25]. Butenko and colleagues proposed that these genomic features may reflect adaptation to alternative hosts or vectors, resulting in altered host-parasite interactions and reduced reliance on certain previously utilized proteins [25].

Within the subgenus *Mundinia*, *L. martiniquensis* and *L. orientalis* constitute the primary human pathogens in Southeast Asia [31,40]. The first autochthonous case of VL in Thailand was reported in 1996 [41]. Several subsequent autochthonous cases were reported under the invalid designation *L*. *siamensis*, a nomen nudum, before this taxonomic confusion was resolved by Leelayoova and colleagues [42], who reclassified these cases into two distinct lineages: the “PG” lineage and the “TR” lineage. The “PG” lineage was later confirmed to be identical to *L. martiniquensis*, originally described in 1995 from cutaneous cases in Martinique [43,44], whereas the clinical isolate corresponding to the “TR” lineage was formally described as *L. orientalis* in 2018 [40]. The identification of these species in both the Caribbean and Southeast Asia has expanded the known geographic and evolutionary range of *Leishmania* (*Mundinia*) parasites [40,44]. Although VL caused by *L. martiniquensis* may resemble VL caused by the *L. donovani* complex, *Mundinia* species differ in phylogenetic position, genome architecture, and probable vector associations [25], indicating that therapeutic evidence derived from classical *Leishmania* complexes should be extrapolated cautiously when evaluating treatment options for Southeast Asian *Mundinia* infections [31].

### 3.2 Epidemiology in Thailand and the region

#### 3.2.1 Clinical spectrum: VL, LCL, DCL, MCL, and HIV co-infection.

Leishmaniasis in Thailand has progressed from sporadic autochthonous reports to an emerging clinical and public health concern. Documented manifestations include visceral leishmaniasis (VL), localized cutaneous leishmaniasis (LCL), diffuse CL (DCL), mucocutaneous leishmaniasis (MCL), and asymptomatic infection [22,24]. Cases have been reported across northern provinces, including Chiang Rai and Lamphun; southern provinces, including Songkhla, Phang Nga, Trang, and Satun; and central provinces, including Bangkok, Lopburi, and Kanchanaburi [23,24,31,45–48]. Autochthonous leishmaniasis currently attributed to *L. martiniquensis* has been documented in Thailand and Myanmar, with Thai cases concentrated mainly in the southern region and Myanmar cases reported in Yangon, encompassing CL, VL, and asymptomatic presentations [31,49,50]. *L. martiniquensis* is the most common species, though *L. orientalis*, *L. infantum*, *L. lainsoni*, *L. major,* and *L. donovani* have also been sporadically detected [23,40,49]. Surveillance remains inconsistent as over 40 autochthonous cases have been identified in Thailand, but only 32 cases (26 VL, 6 CL) are listed in WHO databases [51], indicating probable underreporting and limited case detection [49]. In addition, a cross-sectional study among blood donors in Trang Province detected asymptomatic *Leishmania* infection in 19.0% of participants using direct agglutination testing (DAT) and/or nested PCR (nPCR), with *L. martiniquensis* predominating among nPCR-positive cases, suggesting that clinically recognized cases may underestimate local transmission in endemic areas [22].

Co-infection with HIV is a defining epidemiological feature of leishmaniasis in Thailand, where approximately half of all reported cases occur in PLWH [23,31]. In this population, *Mundinia* infection is associated with relapse, mortality, and concurrent VL and DCL manifestations that may be confused with systemic fungal infections [8,24,47,52]. *L. martiniquensis* can cause VL in immunocompetent hosts, but profound immunosuppression is associated with more severe disease, including VL with concomitant DCL [23,31,46]. MCL caused by *L. martiniquensis* has also been reported in a patient with HIV, demonstrating the parasite’s capacity for mucosal dissemination in the context of immunodeficiency [24,47]. This extensive clinical variability, combined with limited clinical awareness, directly contributes to underdiagnosis and misclassification in the region [24,47,53].

Clinical outcome in leishmaniasis is determined not only by systemic immunosuppression but also by the quality and localization of the host immune response [4,54]. Hyperergic inflammatory responses can drive mucosal or mucocutaneous tissue injury despite relatively low parasite burdens, reflecting pathology in which host-mediated inflammation contributes substantially to tissue damage [4,54,55]. Conversely, anergic or parasite-specific unresponsive states can permit diffuse cutaneous involvement, dissemination, and high parasite burdens with limited local inflammation; this pattern is well recognized in advanced HIV infection but may also occur without generalized immunodeficiency [54,56,57]. These immunopathological poles create distinct therapeutic problems: hyperergic disease requires clinical interpretation of tissue damage beyond parasite burden alone [54], whereas anergic disseminated disease increases the risk of poor drug response, persistent infection, and relapse [46,56,57]. For Southeast Asian *Mundinia* infections, where VL, DCL, and MCL have all been reported, treatment outcomes should therefore be interpreted in the context of both parasite species and host immune phenotype [24,31,46].

#### 3.2.2 Transmission ecology: vectors, reservoirs, and One Health context.

The eco-epidemiology of leishmaniasis in Thailand remains poorly understood, primarily due to limited entomological surveillance that has impeded definitive vector incrimination and full characterization of transmission cycles [31]. Molecular surveys using ribosomal markers (ITS1, SSU rRNA, 3’UTR-HSP70-I) and, more recently, nanopore metabarcoding have detected *L. martiniquensis* and *L. orientalis* DNA in multiple *Culicoides* species, most frequently *C. guttifer*, *C. peregrinus*, and *C. mahasarakhamense* [26,28–30,33,58,59]. Parallel surveillance has detected parasite DNA in several phlebotomine sand flies, including *Sergentomyia* and *Phlebotomus* species [60–63]. Across available Thai entomological surveys, *Leishmania* DNA has been detected more consistently and at higher prevalences in *Culicoides* biting midges than in phlebotomine sand flies; however, this pattern may reflect differences in sampling intensity, trap placement, and microclimate ecology rather than definitive vector status [30,62]. Furthermore, detection rates are highly heterogeneous, with extensive surveys in known endemic provinces yielding entirely negative results, demonstrating the focal and patchy nature of transmission [64,65].

Whether these molecular detections represent true vector competence remains unresolved. *C. peregrinus* has been found naturally infected with promastigote-like flagellates in the foregut of wild-caught specimens, but infective metacyclic forms have not been demonstrated [58]. Recent microscopic dissections of field-caught midges similarly identified *Leishmania* DNA without viable promastigotes, while live *Trypanosoma bennetti*-related trypomastigotes were observed in the same specimens [30]. Laboratory infections of colonized *C. sonorensis* have demonstrated complete *Mundinia* development, metacyclogenesis, and transmission to mammals, whereas non-*Mundinia* species fail to establish mature infections in this midge [27,66]. These findings establish biological plausibility for a midge-mediated transmission cycle but have not yet met Killick-Kendrick’s vector incrimination criteria [67], which require confirmation of infective metacyclic stages in wild vectors and evidence of natural transmission [30].

Putative reservoir hosts further complicate transmission dynamics, with antileishmanial antibodies detected in buffaloes, cattle, cats, and dogs, and *L. martiniquensis* DNA recovered from black rats (*Rattus rattus*) [34,53,68]. These findings indicate exposure of domestic and synanthropic animals to *Mundinia* parasites and identify possible vertebrate hosts that may contribute to local parasite circulation, although reservoir competence remains unproven [28,31]. Reports of *L. martiniquensis* infection in cattle and equines outside Asia indicate a broad mammalian host range, but the relevance of these hosts to transmission in Southeast Asia remains unresolved [69–72]. This pattern is consistent with the broader ecology of zoonotic leishmaniasis, in which animal reservoirs can sustain parasite populations and enable human infection when competent vectors are present [6]. In Thailand, peridomestic agricultural activities, animal enclosures, proximity to termite mounds, and architectural factors such as soil flooring may intensify human-animal-vector contact, creating ecological conditions that favor localized endemicity [22,34].

### 3.3 Current therapeutic limitations

Amphotericin B and azole antifungals remain the primary treatments for leishmaniasis in Thailand; however, disease recurrence following treatment has been documented in multiple patient case reports [24,32,46]. Comparable outcomes have been reported globally, where liposomal amphotericin B monotherapy often fails to achieve a durable cure for VL in immunocompromised cohorts [73,74]. Consequently, current WHO guidelines conditionally recommend combination regimens (S1 Table), particularly liposomal amphotericin B combined with miltefosine, to enhance therapeutic response and reduce relapse rates in HIV-coinfected VL patients in the WHO South-East Asia Region [74]. In Thailand, miltefosine is currently unavailable and is not included in the National List of Essential Medicines, a status that limits routine public sector procurement [32,35,46]. Additional barriers to access include reliance on a single global supplier and historical minimum order batch requirements, while an uncertain market size may further reduce incentives for registration and inclusion in national formularies [19]. The convergence of limited therapeutic options, relapses in immunocompromised patients, incomplete eco-epidemiological characterization, and the absence of miltefosine from national guidelines defines a therapeutic gap that motivates the evaluation of miltefosine as an additional treatment option for Southeast Asia.

## 4. Miltefosine: Clinical evidence and regional therapeutic relevance

### 4.1 Clinical performance across species and regions

Miltefosine’s clinical efficacy varies substantially across *Leishmania* species, geographic regions, clinical forms, and treatment regimens (Table 1). Cure rates approach 90%–95% for VL caused by *L. donovani* in India under controlled conditions [13,75], but decline to 73.3% in Nepal [17] and 59.5% for *L. infantum* VL in Brazil [76]. In CL, outcomes are more variable and less directly comparable across studies because clinical forms, follow-up endpoints, and denominators differ. Cure rates range from 81.6% for *L.* (*Viannia*) *panamensis* in Colombia to 31.3% for *L.* (*V.*) *braziliensis* in Guatemala [77], with intermediate efficacy for *L. tropica* in Pakistan and Iran, at 52.6% and 63.0%, respectively [78,79], and 45.7% for *L. aethiopica* in Ethiopia [80]. DCL caused by *L. mexicana* and *L. amazonensis* responds poorly to miltefosine monotherapy, with cure rates of 0% and 7.7%, respectively [57].

**Table 1:** Summary of miltefosine treatment outcomes by *Leishmania* species, geographic region, and therapy status. Final cure, treatment failure, and relapse rates are reported for VL, CL, and diffuse CL stratified by parasite species, study region, and use of monotherapy versus combination regimens.

*Leishmania* species	Region	Clinical form	Sample size (Year)	Cure (%)	Failure (%)	Relapse (%)	Combination therapy	References
*L.* (*L.*) *donovani*	India	VL	120 (1999)	95	5	5	No	[75]
		VL	299 (2002)	94.3	5.7	3.0	No	[13]
		VL	567 (2012)	90.3	9.7	6.8	No	[18]
	Nepal	VL	120 (2013)	73.3	26.7	20	No	[17]
*L.* (*L.*) *infantum*	Brazil	VL	42 (2019)	59.5	40.5	35.7	No	[76]
*L.* (*L.*) *mexicana*	Guatemala	CL	14 (2004)	64.3	35.7	–	No	[77]
	Venezuela^a^	Diffuse CL	3 (2007)	0	100	100	No	[57]
*L.* (*L.*) *amazonensis*	Venezuela^a^	Diffuse CL	13 (2007)	7.7	92.3	69.2	No	[57]
*L.* (*L.*) *tropica*	Pakistan	CL	76 (2021)	52.6^b^	47.4	–	No^c^	[78]
	Iran	CL	27 (2021)	63.0	37.0	3.7	No^c^	[79]
*L.* (*L.*) *aethiopica*	Ethiopia	CL	94 (2021)	45.7	54.3	22.3	No^c^	[80]
*L.* (*V.*) *panamensis^d^*	Colombia	CL	49 (2004)	81.6	18.4	4.1	No	[77]
*L.* (*V.*) *braziliensis*	Guatemala	CL	16 (2004)	31.3	68.7	–	No	[77]
	Bolivia	CL	44 (2008)	81.8	18.2	9.1	No	[84]
Not designated^e^	India	VL	75 (2022)^f^	85.3	14.7	2.7	No	[73]
		VL	75 (2022)	96	4	1.3	Yes^g^	
Not designated^e^	India	VL	78 (2020)^f^	80.8	19.2	15.4^h^	No	[83]
		VL	66 (2020)	95.5	4.5	0	Yes^i^	

“*Cure*” = sustained clinical and parasitological response at the study-defined final follow-up endpoint (e.g., 6 or 12 months).

“*Failure*” = complement of cure under an intention-to-treat framework, comprising primary non-response, relapse after an initial response, treatment discontinuation due to adverse events, withdrawal or loss to follow-up, or death during treatment or follow-up.

“*Relapse*” = parasitologically confirmed recurrence of disease after an initial cure; reported as a subset of treatment failure and therefore not additive with failure percentages.

“*Combination therapy*” = concurrent administration of miltefosine with another antileishmanial drug.

^a^Species assignment includes one case with contaminated culture; the study reports that three isolates were not definitively identified.

^b^For Kämink and colleagues [78], the cure value shown is 40/76 (52.6%) under the table’s intention-to-treat framework and does not refer to the single-lesion subgroup. The original study reported final cure of 40/52 (76.9%) among patients with assessable final outcomes. Of the remaining participants, 12 defaulted during treatment and 12 were lost to follow-up.

^c^*Miltefosine administered as second-line therapy* indicates prior failure of or contraindication to antimonial treatment.

^d^Colombian cases were from an *L*. *panamensis*-predominant focus; baseline PCR speciation was performed in a subset (7/7 typed isolates were *L.* (*V.*) *panamensis*), and efficacy values reflect the full Colombia intention-to-treat cohort.

^e^Species-level identification was not reported for the enrolled study population; however, these Indian VL cohorts were recruited in settings where *L. donovani* is the predominant reported etiological agent.

^f^This row and the row immediately below it represent separate treatment arms within the same study and are analyzed separately by regimen.

^g^Liposomal amphotericin B, AmBisome, total dose 30 mg/kg in six doses, plus miltefosine 50 mg twice daily for 14 days.

^h^Post-kala-azar dermal leishmaniasis (PKDL) was reported during follow-up but was not included in the relapse calculation.

^i^Liposomal amphotericin B, Fungisome, single dose 7.5 mg/kg, plus miltefosine 2.5 mg/kg/day for 14 days.

In HIV-coinfected VL patients, miltefosine offers a more favorable safety profile than antimonials [81], but therapeutic responses remain heterogeneous, with reduced efficacy reported in Ethiopia [81] and more favorable outcomes in other endemic areas [82]. Combination therapy with liposomal amphotericin B substantially improves outcomes: a recent Indian trial reported cure rates rising from 85.3% with miltefosine monotherapy to 96% with combination treatment in HIV-VL patients [73], and similar improvements (80.8%–95.5%) have been observed in non-HIV-VL cohorts [83]. These findings establish combination therapy as the preferred regimen for immunocompromised VL patients in endemic regions where miltefosine is available.

The species- and form-specific heterogeneity summarized in Table 1 provides a cautionary comparator rather than direct evidence for *L.* (*Mundinia*) treatment in Southeast Asia. No clinical trial data on miltefosine efficacy against *L. martiniquensis* or *L. orientalis* are currently available, and outcomes from non-*Mundinia* species should be interpreted as indirect evidence given the phylogenetic, genomic, and phenotypic divergence of this subgenus [25]. The single documented Thai compassionate-use case, presented in Section 4.2, currently constitutes the only regional clinical evidence of miltefosine activity against a *Mundinia* infection.

### 4.2 First use of miltefosine for *L. martiniquensis* in Thailand: A compassionate-use case

This review includes the first detailed clinical account from Thailand of relapsing diffuse CL and VL caused by *L. martiniquensis* successfully managed using repeated induction courses of combined liposomal amphotericin B and miltefosine. Clinical images from this case were previously published by Jitrukthai and Charatcharoenwitthaya [38], whereas the treatment timeline, parasitological follow-up, and therapeutic interpretation presented here are based on institutional case records and clinical data provided by co-authors involved in the patient’s management.

A 54-year-old patient with HIV/AIDS from Southern Thailand presented with relapsing VL and diffuse CL. The isolate was designated MHOM/TH/2023/CULE10. Despite good adherence to antiretroviral therapy and virological suppression, he experienced multiple relapses following treatment with amphotericin B deoxycholate (1 mg/kg/day for one month, followed by monthly prophylaxis at 50 mg) and subsequently liposomal amphotericin B (5 mg/kg every other day, cumulative dose 30 mg/kg). These relapses manifested as diffuse cutaneous nodules, hepatosplenomegaly, ascites, and pancytopenia, indicating disease recurrence despite prior amphotericin induction and prophylaxis, with further treatment constrained by nephrotoxicity.

In accordance with World Health Organization recommendations and through coordination with the Thai Ministry of Public Health, oral miltefosine (100 mg/day) was provided via compassionate access and administered in combination with liposomal amphotericin B. Each induction with combined therapy resulted in rapid clinical improvement and culture negativity within two weeks, although *Leishmania* DNA remained detectable by qPCR during subsequent relapse episodes. Maintenance therapy with oral miltefosine alone did not sustain remission, with relapse occurring within one month in the context of advanced immunosuppression and limited treatment tolerance, indicating that sustained disease control required continued combination therapy or secondary prophylaxis.

Repeated cycles of combined liposomal amphotericin B and miltefosine achieved remission that was sustained for six months under monthly amphotericin prophylaxis. This case provides clinical evidence that combined miltefosine and liposomal amphotericin B can contribute to sustained remission of refractory *L. martiniquensis* infection in an HIV co-infected patient after amphotericin B monotherapy had failed, although miltefosine monotherapy alone was insufficient to maintain remission. The findings support combination-based induction, molecular confirmation of relapse, and supervised secondary prophylaxis as the operating framework for miltefosine use in *Mundinia* infections in Southeast Asia.

### 4.3 Therapeutic implications of miltefosine for Southeast Asian *Mundinia* leishmaniasis

The clinical evidence summarized above positions miltefosine as a plausible but still insufficiently validated therapeutic addition for Southeast Asian *Mundinia* leishmaniasis. Its oral administration, established use in other endemic settings, and WHO-recommended role in combination regimens for VL-HIV co-infection in East Africa and the WHO South-East Asia Region [74] provide a rationale for consideration in Thailand, where amphotericin B-based therapy remains constrained by toxicity, relapse, and access limitations [24,32,46,47]. However, the absence of clinical trial data for *L. martiniquensis* and *L. orientalis* means that efficacy estimates from *L. donovani*, *L. infantum*, *Viannia*, and other non-*Mundinia* species should be treated as indirect comparators rather than predictive evidence.

The Thai compassionate-use case supports the clinical plausibility of combined miltefosine and liposomal amphotericin B for refractory *L. martiniquensis* infection, particularly in the setting of HIV-associated relapse, but it does not establish generalizable efficacy or define optimal dosing, duration, or secondary prophylaxis. The therapeutic implication is therefore cautious and operational: miltefosine may expand treatment options in carefully selected cases, especially as part of supervised combination therapy, but its introduction should be linked to species-level diagnosis, relapse monitoring, and resistance preparedness. This requirement leads directly to the need to interpret miltefosine’s mechanisms of action and resistance as connected determinants of therapeutic durability.

## 5. Miltefosine’s mechanisms of action and resistance

### 5.1 Clinical emergence of reduced miltefosine susceptibility

Miltefosine efficacy declined after sustained programmatic deployment in the Indian subcontinent, where high initial cure rates against visceral leishmaniasis caused by *L. donovani* were followed by increasing relapse and treatment failure [17,18]. In India, long-term use was associated with a gradual increase in treatment failure compared with earlier trial outcomes, yielding a 90.3% cure rate even under strictly supervised, directly observed therapy in a clinical research setting [18]. In Nepal, relapse approached 20% within 12 months despite completion of therapy (Table 1) [17]. Relapse-associated *L. donovani* isolates from this period generally showed modest *in vitro* susceptibility shifts, often near twofold rather than uniform high-level resistance, indicating that clinical failure cannot be equated automatically with classical resistance phenotypes [85]. This distinction is central for Southeast Asia; while miltefosine remains a rational therapeutic addition for the region, its introduction must be accompanied by comprehensive clinical and parasitological surveillance. As established in other endemic zones, monitoring systems are required to definitively separate clinical relapse, intrinsic drug tolerance, pharmacokinetic failure, host immunological failure, and stable parasite resistance [86].

A terminal elimination half-life of 150–200 hours generates prolonged subtherapeutic exposure when treatment courses are incomplete, producing selection pressure favorable to the emergence of reduced susceptibility [87,88]. Programmatic risk factors compounding this pharmacokinetic vulnerability include high treatment cost, gastrointestinal toxicity, teratogenicity-related contraceptive requirements, unregulated retail availability in some endemic settings, and incomplete adherence to the 28-day oral regimen [9,11,18,19]. These factors are not parasite-intrinsic, but they shape the conditions under which parasite-intrinsic adaptations are selected [19].

Baseline miltefosine susceptibility of *L. martiniquensis* and *L. orientalis* clinical isolates from Southeast Asia has not been comprehensively characterized, and species-resolved susceptibility surveys remain to be performed [36,37,89]. Available experimental data suggest variable baseline susceptibility [36,89], and significantly reduced miltefosine sensitivity has been observed in specific amphotericin B-resistant *L. martiniquensis* lines [37], raising the possibility that resistance trajectories in Southeast Asia may follow molecular paths distinct from those documented in *L. donovani* and *L. infantum* [90]. The Indian subcontinent experience identifies the pharmacokinetic, programmatic, and parasite-intrinsic pressures that any new regional deployment must anticipate [19,88], but it does not predict which mechanisms will dominate in *Mundinia* infections in immunocompromised Southeast Asian patients. Wider miltefosine use in Southeast Asia should therefore be accompanied by species-level diagnosis, baseline susceptibility testing, treatment-response monitoring, and resistance-aware stewardship.

### 5.2 Integrated mechanisms of action and resistance

Miltefosine action and reduced susceptibility converge on the same biological systems: drug transport, membrane lipid organization, mitochondrial stress, redox buffering, cell death execution, and host-parasite interaction. Evidence across these axes comes from functional perturbation studies, laboratory-selected resistant lines, clinical isolates, and omics associations, which differ in causal strength. Table 2 summarizes these mechanisms according to molecular basis, supporting evidence type, species context, and evidentiary strength. Because most mechanistic data derive from non-*Mundinia* species, extrapolation to *L. martiniquensis* and *L. orientalis* requires explicit species-level caution.

**Table 2:** Mechanistic bases of miltefosine resistance in *Leishmania*. Molecular and cellular mechanisms associated with miltefosine resistance in *Leishmania*, including altered drug transport, lipid and membrane remodeling, oxidative stress adaptation, genomic plasticity, and host–parasite interactions. The table summarizes representative molecular bases, the specific tiers of supporting evidence (categorized as clinically resistant isolates, laboratory-selected lines, omics association, functional validation, pharmacological association, and hypothesis), and species contexts. Listed mechanisms should be interpreted as mechanistic capacities rather than predictors of clinical outcome.

Mechanism	Molecular and cellular basis	Representative findings	Evidence type	*Leishmania* species	References
Altered drug transport (uptake)	Loss of function of the miltefosine P-type ATPase transporter (MT)	(1) Inactivating point mutations, resulting in amino acid substitutions in MT (e.g., T420N, L856P, G852E, L832F), deletions, or frameshifts reduce intracellular miltefosine accumulation; (2) genetic complementation with wild-type MT restores susceptibility.	(1) Clinically resistant isolates; laboratory-selected lines; omics association (2) Functional validation	*L. donovani*, *L. major*, *L. amazonensis*, *L. infantum*	[91–95]
	Impaired MT trafficking due to Ros3 (CDC50/LEM3) dysfunction	(1) Reduced Ros3 expression or copy number is linked to reduced susceptibility in *L. braziliensis* and *L. tropica*, which is reversible by overexpression. (2) In contrast, clinical *L. donovani* and *L. infantum* isolates commonly retain wild-type Ros3 levels, indicating alternative resistance mechanisms in field settings.	(1) Clinically resistant isolates; laboratory-selected lines; comparative species observation; omics association; functional validation (2) Clinically resistant isolates	*L. tropica*, *L. braziliensis*, *L. donovani*, *L. infantum*	[10,94,96–100]
Altered drug transport (efflux)	ABC transporter-associated reduction in intracellular drug accumulation	(1) Overexpression of ABC transporters (e.g., LiABCG4, LiABCG6, LtrMDR1) is associated with reduced intracellular miltefosine accumulation in experimental models. (2) Transcriptional responses are species-, strain-, and stage-specific and are frequently absent in clinical isolates.	(1) Laboratory-selected lines; functional validation; pharmacological association (2) Clinically resistant isolates; laboratory-selected lines; omics association	*L. tropica*, *L. infantum*, *L. donovani*	[96,101–104]
Lipid metabolism and membrane alterations	Membrane lipid remodeling affecting phospholipid and sterol composition	(1) Acute miltefosine exposure depletes phosphatidylcholine with compensatory increases in sphingolipids and sterols. (2) Stable resistance involves constitutive membrane remodeling, including increased fatty acid saturation, altered sterol composition, and differential expression of lipid metabolism genes.	(1) Omics association (2) Laboratory-selected lines; omics association; functional validation; pharmacological association	*L. donovani*, *L. infantum*, *L. major*	[104–108]
Oxidative stress response	Enhanced antioxidant capacity and redox metabolic reprogramming	(1) Resistant *L. donovani* and *L. tropica* upregulate antioxidant enzymes (e.g., FeSODA, SIR2, TXNPx, PRX1A), enhancing detoxification of drug-induced ROS. (2) In *L. donovani*, resistance is further supported by increased pentose phosphate pathway (PPP) flux and NADPH production, (3) whereas responses in *L. infantum* are variable.	(1) Clinically resistant isolates; laboratory-selected lines; omics association; functional validation (2) Functional validation; pharmacological association; hypothesis (3) Laboratory-selected lines; omics association	*L. donovani* (enzymes + PPP); *L. tropica* (enzymes); *L. infantum* (variable)	[96,109–113]
Genomic plasticity	Structural genomic variation affecting drug transport and membrane homeostasis	(1) Deletion of the miltefosine susceptibility locus, including *NUC1* and *NUC2*, is associated with lipid remodeling and reduced drug toxicity in *L. infantum*. (2) Under drug pressure, parasites also select for MT-inactivating mutations and aneuploidy that reduce effective transporter dosage.	(1) Clinically resistant isolates; omics association; functional validation (2) Clinically resistant isolates; laboratory-selected lines; omics association	*L. infantum*, *L. major*, *L. donovani*	[93,94,98,114–116]
Cell death modulation	Attenuation of mitochondrial dysfunction and apoptosis-like pathways	(1) Miltefosine induces mitochondrial depolarization and apoptosis-like death in susceptible parasites. (2) Resistant *L. donovani* and *L. tropica* rely on the upregulation of antioxidative enzymes and stress-response proteins to preserve mitochondrial functions. (3) In *L. infantum*, adaptive responses to miltefosine exhibit intra-species heterogeneity: an *in vitro*-selected resistant line sustains bioenergetics through metabolic remodeling with decreased canonical ROS detoxification [113], whereas a clinically resistant isolate demonstrates significant upregulation of canonical ROS detoxification enzymes [117].	(1) Pharmacological association (2) Clinically resistant isolates; laboratory-selected lines; omics association; functional validation; pharmacological association (3) Clinically resistant isolates; laboratory-selected lines; omics association	*L. donovani*, *L. tropica* (antioxidant defense), *L. infantum* (metabolic remodeling)	[109,110,112,113,117,118]
Protein processing and energy metabolism	Stress-response activation and reconfiguration of energy metabolism	(1) Resistance is accompanied by the induction of stress-response proteins (HSP60, HSP70, HSP83, STI1, PCNA) and (2) reprogramming of energy metabolism, including increased components of oxidative phosphorylation and altered lipid utilization	(1) Clinically resistant isolates; laboratory-selected lines; omics association (2) Laboratory-selected lines; omics association	*L. tropica*, *L. donovani*, *L. infantum*	[96,104,113,117,118]
Host–parasite interaction and clinical relevance	Parasite-mediated modulation of host immune responses and fitness	(1) Clinical relapses often occur without high-level intrinsic resistance. (2) Instead, treatment failure correlates with parasite-mediated host immune modulation, including reduced nitric oxide production or increased IL-10/TNF-α ratios. (3) Fitness effects are context-dependent, ranging from growth defects to enhanced infectivity.	(1) Clinically resistant isolates (2) Clinically resistant isolates; functional validation; hypothesis (3) Clinically resistant isolates; laboratory-selected lines; functional validation	*L. tropica*, *L. donovani*, *L. infantum*	[94,96–98,115,118,119]
Endosymbiotic viruses	Virus-driven host immune dysregulation independent of intrinsic drug susceptibility	(1) LRV1-positive *L. braziliensis* and *L. guyanensis* infections are associated with treatment failure and relapses despite preserved *in vitro* drug susceptibility, implicating host immune dysregulation rather than intrinsic resistance, although this association is not universal across cohorts and has not been demonstrated for LRV2-positive *L. major* isolates. (2) Viral particles are transmitted via parasite-derived exosomes. (3) Experimental viral depletion reduces parasite infectivity in *L. martiniquensis* and parasite proliferation in *L. major*, while effects on antileishmanial drug response remain untested.	(1) Clinically resistant isolates (2) Omics association; hypothesis (3) Functional validation; pharmacological association; hypothesis	*L. guyanensis, L. braziliensis, L. panamensis, L. naiffi* (LRV1); *L. major, L. aethiopica, L. infantum, L. tropica* (LRV2); *L. martiniquensis* (*Lmar*LBV1)	[120–129]

#### 5.2.1 Translocation dynamics: active uptake and efflux.

**Fig 1:**
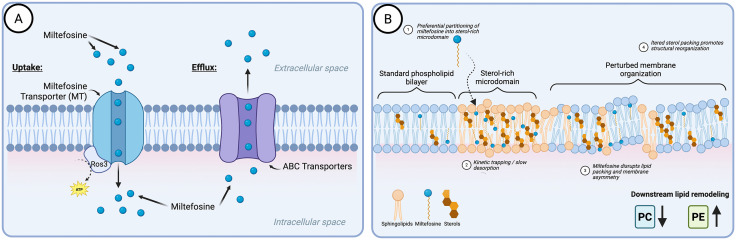
Membrane-associated mechanisms of miltefosine uptake, efflux, and lipid remodeling in *Leishmania.* **(A)** Miltefosine accumulation depends on active inward translocation across the plasma membrane through the miltefosine transporter (MT), a P4-ATPase that functions with the Ros3 β-subunit. ABC transporters can reduce intracellular drug retention by promoting outward transport or efflux, although their contribution varies by species, strain, and resistant background. **(B)** After membrane association, miltefosine partitions into sterol-rich microdomains, where physicochemical intercalation, kinetic retention, and altered sterol packing can perturb membrane organization [144,145]. These membrane effects are associated with disrupted lipid packing, altered phospholipid asymmetry, and downstream lipid remodeling, including reduced phosphatidylcholine (PC) and increased phosphatidylethanolamine (PE). The figure presents a generalized model synthesized from studies in non-*Mundinia* species and model membrane systems; direct validation of these processes in *L. martiniquensis* and *L. orientalis* remains limited. Created in BioRender. Lerona, P.G. (2026) https://BioRender.com/u6l8rje.

In *L. donovani* and *L. infantum*, plasma membrane inward translocation via the miltefosine transporter (MT) complex constitutes the primary determinant of intracellular drug accumulation and the most frequently disrupted target in highly resistant parasites [10,130]. MT, a P4-ATPase [95], functions cooperatively with its accessory β-subunit Ros3 [130] to mediate the ATP-dependent internalization of specific phospholipids and alkylphosphocholines (Fig 1A). In *L. donovani*, fluorescent alkylphosphocholine analogues accumulate to intracellular concentrations approximately 100-fold higher than the extracellular environment, consistent with active inward translocation rather than passive partitioning [131]. Functional complementation studies establish MT/Ros3 as the principal route of drug entry, since episomal restoration of wild-type MT or Ros3 re-establishes susceptibility in transporter-deficient *L. infantum* parasites [94,132]. Furthermore, metabolomic profiling of resistant *L. infantum* shows absent intracellular drug accumulation and the absence of the metabolic collapse otherwise observed in susceptible parasites, consistent with impaired uptake as the proximal resistance event [133].

Loss-of-function changes in MT or Ros3 reduce miltefosine accumulation and constitute the best-validated resistance mechanism described to date [10,94,134]. Laboratory-selected resistant lines frequently acquire genetic lesions that disrupt this transport axis, employing inactivating missense point mutations [10,104,135] as well as nonsense mutations [10,135]. Whole-genome sequencing of laboratory-selected *L. major* miltefosine-resistant populations identifies multiple independent point mutations and small deletions in MT, demonstrating polyclonal mutational heterogeneity wherein individual clones within a single selection differ in both genotype and susceptibility phenotype [136]. Functionally validated alterations include a T420N substitution within the ATPase phosphorylation motif in *in vitro*-selected *L. donovani* [95,104], CRISPR-Cas9 disruption producing large structural deletions [137], an L832F substitution in a clinical *L. infantum* isolate [92], and frameshift mutations affecting LiMT and LiROS3 confirmed as causal by complementation [94,132]. Intrinsic tolerance in *L. braziliensis* arises from limited LbRos3 dosage that restricts plasma membrane localization of LbMT [100]. Clinical isolate observations qualify the laboratory picture: relapse-associated *L. donovani* isolates from India frequently retain wild-type MT and Ros3 sequences alongside baseline expression levels [97]. Additionally, aneuploidy-mediated dosage modulation involving the reduced copy number of chromosome 13 (encoding LdMT) provides an adaptive route under experimental drug pressure, as documented during the *in vitro* laboratory selection of Nepalese clinical strains [115,138]. Gain-of-function screening through Cos-Seq intrinsically excludes MT/Ros3 loss-of-function but identifies alternative resistance determinants linked to sterol biosynthesis and membrane lipid homeostasis [106], indicating that transporter-independent routes can also confer resistance.

ATP-binding cassette (ABC) transporters contribute to reduced intracellular drug retention through enhanced efflux [10,139]. Increased expression of ABCG4 and ABCG6 at the plasma membrane and flagellar pocket of laboratory-selected *L. infantum* and *L. tropica* lines correlates with reduced intracellular miltefosine retention and cross-tolerance to other alkylphospholipids [96,101,102], while independent *L. donovani* models demonstrate upregulation of different ABC classes, such as ABCG2, ABCG5, and ABCA7 [104]. Functional studies show that ATP hydrolysis is required for the efflux phenotype, since catalytic inactivation of ABCG6 abolishes the tolerance conferred by overexpression [96]. Pharmacological inhibition studies support an efflux contribution: beauvericin partially restored miltefosine susceptibility in *L. tropica* [96], and sitamaquine reversed miltefosine resistance through indirect modulation of ABC-dependent efflux rather than competitive transport [103]. The efflux contribution is not conserved across resistant backgrounds, however, as independent miltefosine-resistant *L. donovani* lines instead show downregulation of MDR1 and ABCG4 [140]. Furthermore, while increased ABCB2 (TAP1) abundance occurs in some miltefosine-resistant *L. infantum* clinical isolates [141], it is often observed alongside extensive remodeling of stress-response pathways rather than as an isolated dominant transport signature [117], and overexpression of P-glycoprotein-like transporters in arsenite-selected *L. donovani* does not confer cross-resistance to miltefosine [142]. Proteomic and transcriptomic associations with ABC transporter expression in clinical isolates therefore indicate variable and conditional involvement rather than a universal mechanism. Extracellular vesicle-mediated changes in cargo composition and morphology have been characterized in drug-resistant *L. infantum*. Miltefosine-resistant parasites produce larger extracellular vesicle subpopulations and exhibit a proteomic signature distinct from wild-type parasites and from antimony- or amphotericin B-resistant lines [143]. These changes suggest an auxiliary stress-response or remodeling phenotype, but their functional contribution to miltefosine resistance remains unresolved.

MT/Ros3 localization, transporter expression, and ABC efflux activity under miltefosine pressure remain unresolved in *L. martiniquensis* and *L. orientalis*. Candidate transporter variation has been described in amphotericin B-resistant *L. martiniquensis*, but its contribution to reduced miltefosine susceptibility requires functional testing [90].

#### 5.2.2 Membrane intercalation and lipid remodeling.

Acute miltefosine exposure depletes phosphatidylcholine (PC) and increases phosphatidylethanolamine (PE) in *L. donovani*, producing a marked reduction of the PC/PE ratio and impaired membrane homeostasis, with elevated lysophospholipid levels consistent with enhanced phospholipid remodeling and membrane stress [107] (Fig 1B). These changes are attributed to disruption of Kennedy pathway activity (S1 Fig), particularly methylation-dependent steps and possibly cytidyltransferase activity, rather than simple substrate deprivation [107]. Although miltefosine inhibits choline transport in *L. major*, promastigotes of this species are not strict choline auxotrophs, indicating that reduced choline uptake alone is unlikely to explain parasite killing [146]. Short-term exposure also induces sterol accumulation, including increased ergosterol abundance, and disrupts sphingolipid metabolism, with sphingosine and ceramide elevation reported in *L. donovani* and *L. major* [105]. In *L. mexicana*, inhibition of alkyl-specific acyl-CoA acyltransferase has been described at concentrations exceeding antiproliferative thresholds, suggesting a secondary or context-dependent contribution [147]. Comparative preclinical and clinical studies show broad but variable miltefosine activity across CL-causing species, supporting a membrane-active mechanism with species-specific differences in potency and response [148,149].

In *L. donovani*, stably resistant promastigotes adapt primarily through reduced unsaturation of fatty-acid alkyl chains, decreased membrane fluidity, depleted C24-alkylated sterols, and increased incorporation of exogenous cholesterol [150]. Genetic and functional studies corroborate lipid metabolism as a determinant of reduced susceptibility: mutations altering *L. infantum* fatty-acid elongase (LinJ.14.0790) and long-chain acyl-CoA ligase (LinJ.13.0300) increase tolerance [135], while overexpression of lipase precursor-like protein (LinJ.31.0870) in *L. donovani* enhances resistance by promoting fatty-acid utilization and mitigating oxidative stress rather than altering drug transport [118]. In *L. major*, serine palmitoyltransferase-deficient promastigotes accumulate cholesterol, reduce ergosterol levels, and show diminished miltefosine susceptibility despite preserved drug uptake [105]. Loss of the miltefosine susceptibility locus containing *NUC1* and *NUC2* in *L. infantum* also increases basal sterol and glycerophospholipid content, consistent with membrane sequestration limiting effective drug action [98]. These findings indicate that membrane remodeling can reduce susceptibility, although the extent to which such changes act as primary resistance drivers rather than compensatory adaptations remains unresolved.

*L. martiniquensis* provides the clearest regional indication that sterol remodeling may intersect with miltefosine susceptibility. An *in vitro*-selected amphotericin B-resistant line and a clinical relapse isolate both exhibited elevated amphotericin B IC_50_ values together with reduced miltefosine susceptibility [37]. Genome analysis identified a stop-gained mutation in sterol C-24 reductase alongside missense mutations affecting an ABC transporter-like protein [90]. These findings make sterol and membrane remodeling plausible contributors to cross-resistance, but functional validation and clinical cohort evidence remain lacking. This cross-resistance phenotype is considered further in Section 5.3.

#### 5.2.3 Intracellular ion homeostasis, mitochondrial toxicity, and redox buffering.

**Fig 2:**
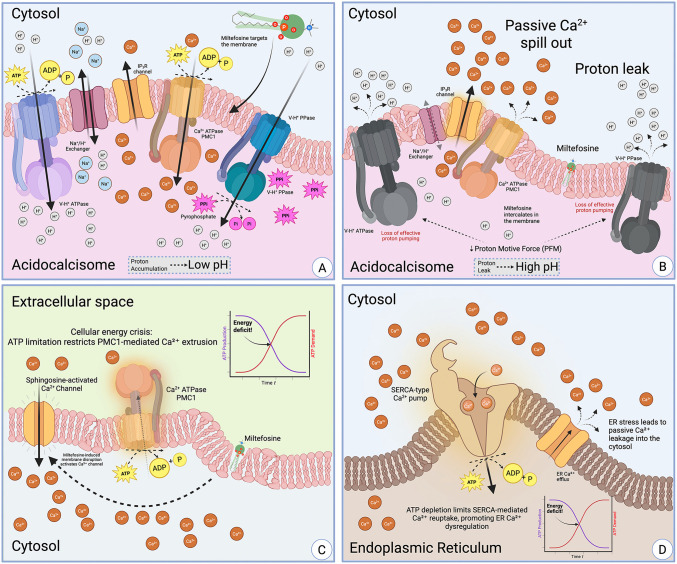
Disruption of Ca^2+^ homeostasis by miltefosine in *Leishmania.* **(A)** Under basal conditions, acidocalcisomes maintain ionic balance through the coordinated activity of the vacuolar H⁺-ATPase, vacuolar H⁺-pyrophosphatase, and PMC1, a Ca^2+^-transporting ATPase, sustaining a proton motive force and an acidic lumen that supports Ca^2+^ sequestration. **(B)** Miltefosine-associated membrane perturbation can compromise proton retention, leading to proton leakage, loss of proton motive force, and passive Ca^2+^ release from acidocalcisomes into the cytosol, without implying direct inhibition of PMC1. **(C)** At the plasma membrane, miltefosine enhances Ca^2+^ influx through sphingosine-activated Ca^2+^ channels, while ATP depletion may limit PMC1-mediated Ca^2+^ extrusion, promoting cytosolic Ca^2+^ accumulation. **(D)** Increased cytosolic Ca^2+^ and ATP limitations may impair sarco/endoplasmic reticulum Ca^2+^-ATPase-mediated reuptake, contributing to endoplasmic reticulum Ca^2+^ dysregulation and organellar stress. This schematic represents a generalized model of miltefosine-associated Ca^2+^ disruption based mainly on studies in non-*Mundinia* species and related kinetoplastid calcium biology. Created in BioRender. Lerona, P.G. (2026) https://BioRender.com/t9n5kgx.

In *L. donovani*, miltefosine activates a sphingosine-dependent plasma membrane Ca^2+^ channel, leading to rapid acidocalcisome alkalinization and cytosolic Ca^2+^ overload [151] (Fig 2). Subsequent mitochondrial Ca^2+^ uptake accelerates electrochemical depolarization [152], while Ca^2+^ overload and mitochondrial dysfunction are expected to enhance reactive oxygen species production and contribute to bioenergetic collapse in kinetoplastids [153]. Miltefosine also directly inhibits mitochondrial cytochrome *c* oxidase, or complex IV, producing severe mitochondrial depolarization, ATP depletion, and metabolic collapse in *L. donovani* promastigotes [154]. Comparative kinetoplastid data support a mitochondrial contribution to drug action: bloodstream *Trypanosoma brucei* lacks a functional cytochrome-dependent electron transport chain [155], and this respiratory configuration is associated with lower susceptibility to miltefosine-mediated mitochondrial toxicity [154]. The relative order of membrane perturbation, complex IV inhibition, and redox collapse as initiating events remains unresolved [156]. Calcium imbalance should now be considered an additional candidate upstream stressor based on later work, but its position within this sequence remains to be established [151,152].

Quantitative proteomics of *in vitro*-selected miltefosine-resistant *L. infantum* revealed increased abundance of respiratory chain components across complexes III, IV, and V, including F_0_F_1_-ATP synthase, together with enrichment of fatty-acid β-oxidation enzymes, a pattern interpreted as maintenance of electron transport and ATP generation under drug pressure [113]. A multidrug-refractory mucosal *L. braziliensis* isolate showed elevated ATPase α subunit and mtHSP70 expression, indicating that mitochondrial stress-response proteins may also be altered in refractory clinical infection [157]. These changes may reflect mitochondrial adaptation in reduced-susceptibility contexts, but their causal contribution remains unresolved because most evidence is associative rather than functionally validated.

Iron superoxide dismutase A (FeSODA) is upregulated in miltefosine-resistant *L. donovani* and contributes to detoxification of drug-induced superoxide radicals [158,159]. Elevated cytosolic and mitochondrial tryparedoxin peroxidases also contribute to peroxide detoxification and redox buffering [110]. Proteomic profiling identified peroxiredoxin as a shared stress-response protein across *L. infantum* parasites resistant to miltefosine, antimonials, or paromomycin, consistent with generalized adaptation to oxidative stress rather than drug-specific selection [141]. The functional contribution of individual redox enzymes is context-dependent: partial downregulation of FeSODA in *L. infantum* was paradoxically associated with increased resistance, attributed to compensatory induction of alternative antioxidant pathways, including ascorbate peroxidase [160]. Activation of the pentose phosphate pathway in *L. donovani*, with overexpression of glucose-6-phosphate dehydrogenase and transaldolase, increases NADPH availability and confers resistance to multiple drugs, including miltefosine, amphotericin B, and antimony, though not paromomycin [111]. Stress-response and chaperone proteins further modulate mitochondrial integrity: HSP83 enrichment in an antimony-resistant *L. donovani* clinical isolate prevented mitochondrial membrane depolarization, and HSP83 transfection conferred miltefosine resistance in otherwise susceptible parasites [161]. Overexpression of LdTCP1γ increased the miltefosine IC_50_ approximately 1.8-fold through increased thiol buffering and induction of tryparedoxin peroxidase [162]. Differential abundance of these proteins in resistant clinical isolates does not by itself establish causal contribution. Comparable calcium responses, mitochondrial vulnerabilities, redox-buffering pathways, and resistance-associated proteomic adaptations have not been defined in *L. martiniquensis* or *L. orientalis*.

#### 5.2.4 Cell death-associated phenotypes and survival adaptation.

**Fig 3:**
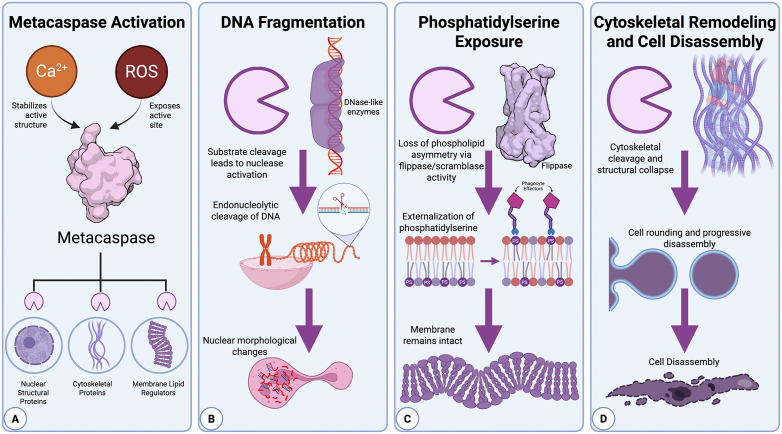
Apoptosis-like cell death pathways in *Leishmania* under miltefosine stress. Schematic representation of major phenotypic hallmarks associated with miltefosine-induced apoptosis-like death. **(A)** Metacaspase activation associated with Ca^2+^ and ROS stress, contributing to proteolytic processing of nuclear, cytoskeletal, and membrane-associated proteins. **(B)** DNA fragmentation through endonuclease-mediated cleavage accompanied by nuclear condensation. **(C)** Loss of phospholipid asymmetry resulting in phosphatidylserine externalization while plasma membrane integrity is preserved. **(D)** Cytoskeletal remodeling and progressive cell disassembly culminating in parasite death. These features resemble metazoan apoptosis phenotypically but are mediated through parasite-specific, non-canonical molecular machinery and should not be interpreted as evidence of canonical caspase-dependent apoptosis. Created in BioRender. Lerona, P.G. (2026) https://BioRender.com/q3d7unm.

In susceptible *L. donovani* and *L. amazonensis*, miltefosine exposure induces phosphatidylserine externalization with preserved plasma membrane integrity, accompanied by cell shrinkage, oligonucleosomal DNA fragmentation, and accumulation of parasites in the sub-G0/G1 population [163–166] (Fig 3). These features have often been interpreted as apoptosis-like cell death [165], but the classification of programmed cell death in protozoa remains debated because several hallmarks may also occur during incidental or non-regulated death pathways [153]. Canonical caspases are absent in *Leishmania*, although metacaspases undergo stimulus-dependent auto-processing during oxidative stress and drug exposure, and endonuclease G translocates from the mitochondrion to the nucleus to mediate oligonucleosomal DNA fragmentation [167–169]. In *L. donovani*, methionine aminopeptidase 2 inhibition prevents ΔΨm collapse, cytosolic Ca^2+^ accumulation, and DNA fragmentation during miltefosine-induced stress, but does not ultimately prevent parasite death [170].

kDNA disruption during miltefosine exposure is best interpreted as a downstream mitochondrial injury phenotype rather than a primary drug target. In *L. donovani*, dyskinetoplastidy (loss or disintegration of the kinetoplast) follows mitochondrial membrane potential collapse, and kDNA loss is attributed to mitochondrial permeabilization and nuclease access rather than direct inhibition of kDNA maintenance [142]. Drug-induced stress can also activate autophagy as an early survival response, although prolonged activation may converge with apoptosis-like pathways and promote parasite death [171].

Resistant *L. donovani* lines preserve mitochondrial function through upregulation of antioxidative enzymes that suppress oxidative stress-associated apoptosis-like death [109,110,112]. Evidence from *L. infantum* indicates mechanistic heterogeneity: an *in vitro*-selected resistant line sustained bioenergetics through metabolic remodeling rather than canonical ROS detoxification [113], whereas a refractory clinical isolate showed increased abundance of antioxidative enzymes and stress-response proteins, including HSP60, STI1, and PCNA [117]. Resistant *L. tropica* similarly shows induction of stress-response and DNA repair proteins, including HSP60, HSP70, HSP83, and PCNA, alongside reprogramming of energy metabolism [96]. Kinetoplastid calpain-related proteins further illustrate context-dependent regulation: SKCRP14.1 transfection in *L. donovani* increased miltefosine tolerance while sensitizing parasites to antimony [161]. Functional evidence supports causal roles for selected effectors such as SKCRP14.1 and HSP83 [161], but many reported stress-response signatures derive from comparative omics analyses of clinical isolates or laboratory-selected lines. Differential protein abundance should therefore be interpreted as associative unless supported by direct perturbation experiments. Comparable cell death-associated phenotypes, kDNA disruption, autophagic responses, and survival adaptations under miltefosine pressure have not been characterized in *L. martiniquensis* or *L. orientalis*.

#### 5.2.5 Host immunomodulation and host-parasite determinants of treatment outcome.

Miltefosine immunomodulation has been systematically reviewed by Palić and colleagues [172], and only host-mediated effects directly relevant to treatment outcome and resistance interpretation are considered here. In *L. donovani*-infected macrophages, miltefosine restores IFN-γ responsiveness by increasing IFN-γ receptor expression, promoting STAT1 phosphorylation, and counteracting SHP-1 phosphatase activity [173]. The same study showed induction of inducible nitric oxide synthase and nitric oxide production through PKC- and PI3K-dependent p38 MAPK signaling [173]. Miltefosine also upregulates TLR4 and TLR9 in *L. donovani*-infected macrophages, amplifying IL-12, TNF-α, nitric oxide, and downstream IFN-γ production [174]. At the parasitophorous vacuole membrane, miltefosine disrupts host Akt activation that normally supports intracellular persistence in *L. donovani* and *L. amazonensis* infection models [175].

Miltefosine enhances nitric oxide production in *L. amazonensis*-infected macrophages but reduces nitric oxide production in uninfected macrophages, indicating that host immune activation depends on the infected cellular state rather than reflecting uniform pro-inflammatory stimulation [176]. In human PBMCs infected with *L. panamensis*, miltefosine suppresses IL-10 and IL-13 without significantly altering IFN-γ or TNF-α production [177]. This context dependence is clinically relevant in immunosuppressed patients, including those with HIV co-infection, where impaired cellular immunity may reduce the contribution of host-mediated parasite clearance and partly explain heterogeneous outcomes and the need for combination regimens to achieve durable cure [73] (Section 4.1).

Miltefosine-resistant parasites may modify macrophage infection and cytokine balance through resistance-associated metabolic adaptations. In *L. donovani*, altered MAPK expression has been associated with miltefosine resistance and may influence stress adaptation and life-cycle regulation [178]. A lipase precursor-like protein associated with miltefosine tolerance enhances macrophage infectivity and skews cytokine responses toward anti-inflammatory profiles, linking metabolic adaptation with host-cell persistence [118]. In a murine model, an experimentally selected miltefosine-resistant *L. infantum* strain triggered enhanced early innate immune activation in the liver, with increased NK and NKT cell activity and higher systemic IFN-γ; miltefosine treatment partially restored parasite fitness *in vivo*, indicating a drug-dependent rather than constitutive immune-evasion phenotype [179]. Comparable resistance-associated host-parasite fitness effects have not been characterized in *L. martiniquensis* or *L. orientalis* under miltefosine pressure.

In *L. guyanensis*, the endosymbiotic *Leishmania* RNA virus 1 (LRV1) is detected by host TLR3 and triggers proinflammatory signaling that supports parasite persistence and metastatic spread [180]. Clinical studies associate LRV1-positive infections with treatment failure or relapse in *L. guyanensis* [124], *L. braziliensis* [121], and *L. naiffi* [129] despite preserved *in vitro* drug susceptibility, although this association is not universal and one cohort of *L. guyanensis* isolates showed no correlation between LRV1 status and pentamidine treatment failure [125], indicating that viral effects may modulate therapeutic outcome independently of intrinsic parasite resistance without uniformly determining it. LRV1 suppresses inflammasome activation by inhibiting NLRP3 through TLR3-mediated autophagic degradation [181] and by interfering with non-canonical caspase-11 activation [182,183], reducing effective microbicidal responses despite increased inflammation, while packaging within parasite-derived extracellular vesicles promotes viral persistence [122]. In contrast, the immunomodulatory effects of *Leishmania* RNA virus 2 (LRV2) in *L. major* appear distinct; while LRV2 is associated with reduced IL-1β expression, it significantly upregulates *NLRP3* gene expression at later infection stages *in vitro* [127], indicating divergent or species-specific viral-host interactions. The leishbunyavirus *Lmar*LBV1, the first non-LRV RNA virus identified in *Leishmania*, was detected in a Martinique isolate of *L. martiniquensis* [126]. Experimental viral depletion in this isolate reduces parasite infectivity in murine macrophages, supporting a direct role for viral endosymbionts in host-parasite interactions, although effects on antileishmanial drug susceptibility remain untested [126]. Viral endosymbionts should therefore be interpreted as host-parasite modifiers of treatment outcome rather than proven miltefosine resistance mechanisms. The distribution, functional effects, and clinical relevance of viral endosymbionts in Southeast Asian *Mundinia* infections require direct investigation.

### 5.3 Cross-resistance with amphotericin B in *L. martiniquensis*

Both an *in vitro*-selected amphotericin B-resistant line and a clinical relapse isolate of *L. martiniquensis* exhibit elevated amphotericin B IC_50_ values together with reduced miltefosine susceptibility, indicating a cross-resistance phenotype between these membrane-targeting drugs under experimental and relapse-associated contexts [37]. Genome analysis of resistant lines identified a stop-gained mutation in sterol C-24 reductase, disrupting the terminal step of ergosterol synthesis (S2 Fig), alongside additional mutations affecting an ABC transporter-like protein, LSCM1_01856 [90]. These findings make sterol remodeling and altered membrane organization plausible contributors to reduced miltefosine susceptibility, potentially through mechanisms independent of canonical MT/Ros3 transporter disruption.

Amphotericin B formulations have been the therapeutic mainstay for autochthonous leishmaniasis in Thailand [24,32], and sustained amphotericin B exposure could plausibly select parasite phenotypes with reduced miltefosine susceptibility before wider miltefosine deployment. Current evidence remains restricted to *in vitro* selection and one relapse isolate, and clinical cross-resistance has not been demonstrated in patient cohorts. The convergence of amphotericin B reliance, emerging *Mundinia* infection, and experimentally observed reduced miltefosine susceptibility supports prospective phenotypic and genotypic monitoring of clinical isolates from amphotericin B-treated patients in Thailand.

### 5.4 Knowledge gaps in *Mundinia*

Miltefosine resistance models remain insufficiently defined for *Mundinia* in Southeast Asia. The major mechanistic axes described in this section derive largely from *L. donovani*, *L. infantum*, *L. major*, *L. mexicana*, or other non-*Mundinia* species. Direct *Mundinia* evidence is limited to reduced miltefosine susceptibility in amphotericin B-resistant *L. martiniquensis* [37,90] and the identification of *Lmar*LBV1 as a host-parasite modifier in *L. martiniquensis* [126]. These observations support focused mechanistic studies of *Mundinia*, but they do not establish which resistance pathways will emerge under miltefosine exposure in regional clinical isolates.

Mechanistic interpretation of miltefosine response in Southeast Asia requires baselines that are currently unavailable for *Mundinia*. Species-resolved susceptibility surveys are needed for *L. martiniquensis* and *L. orientalis* to distinguish natural variation from emerging reduced susceptibility. Functional studies should test whether resistance axes defined in other *Leishmania* species, including MT/Ros3 transport, ABC efflux, lipid and sterol remodeling, mitochondrial and redox adaptation, cell death tolerance, and host-parasite fitness effects, operate in *Mundinia*. The amphotericin B-associated cross-resistance signal described above should be incorporated into future *Mundinia* susceptibility baselines. These unresolved questions define the stewardship conditions needed to introduce miltefosine as a useful but carefully monitored therapeutic option in Southeast Asia.

## 6. Diagnostics, surveillance, and resistance preparedness

Resistance preparedness for miltefosine in Southeast Asia requires two technical foundations: species-level diagnosis and longitudinal susceptibility surveillance. In Thailand, current diagnostic capacity supports case confirmation but remains insufficient for establishing the species-resolved baseline needed to interpret future shifts in drug response.

### 6.1 Diagnostic capacity in Thailand

Diagnosis of leishmaniasis in Thailand relies on parasitological, serological, and molecular approaches, each constrained by sensitivity, species resolution, or operational feasibility. Microscopic examination of stained smears and parasite culture retains high specificity but is limited by low sensitivity, prolonged turnaround times, and dependence on specialized expertise, with culture capacity restricted to a small number of laboratories [50,184]. Serological assays widely deployed in other endemic regions, including enzyme-linked immunosorbent assay (ELISA), indirect fluorescent antibody testing (IFAT), direct agglutination test (DAT), and the rK39 rapid test, remain poorly validated for autochthonous Thai *Leishmania* species and exhibit uncertain diagnostic performance in local settings [50,184]. These constraints have driven increased reliance on PCR-based diagnostics, which combine high analytical sensitivity and specificity with the capacity to detect parasite DNA from non-invasive samples such as saliva and buccal swabs, supporting earlier identification of infection in immunocompromised patients [50,185,186].

Species-level identification is particularly important in Thailand because *L. martiniquensis* and *L. orientalis* are associated with distinct clinical manifestations and may require different treatment approaches [186]. Conventional PCR assays targeting internal transcribed spacer 1 (ITS1) and heat shock protein 70 (HSP70) loci are widely applied for species identification, and a duplex TaqMan-based quantitative PCR has been developed to permit simultaneous detection and quantification of *L. martiniquensis* and *L. orientalis* in clinical samples [186,187]. Point-of-care platforms, including loop-mediated isothermal amplification and colorimetric assays, have been evaluated as rapid screening tools, although definitive species assignment continues to depend on sequencing-based confirmation [184,188]. Diagnostic infrastructure for asymptomatic carriage screening, vector-host investigation, and population-level surveillance remains unevenly distributed across Thai endemic areas, and several routinely available platforms have not been validated for the *Mundinia* species relevant to autochthonous transmission.

### 6.2 Genomic surveillance and parasitomics

Parasitomic approaches extend molecular diagnostics into surveillance by combining species identification with population-level genetic characterization [189]. Amplicon-based sequencing methods, ranging from Sanger sequencing to nanopore-enabled metabarcoding, have been applied to characterize *Leishmania* haplotype diversity, vector blood-meal sources, and transmission ecology in Thailand, supporting the integration of human, animal, and entomological surveillance [26,28–30]. These platforms generate species-resolved genomic data that can be used to track parasite population structure over time, identify cross-border movement of strains, and provide reference data against which future susceptibility studies can be calibrated.

Genomic surveillance does not directly detect emerging clinical resistance, but species-resolved sequencing enables monitoring of molecular markers associated with reduced susceptibility in well-characterized species, which may inform treatment decisions and stewardship policy when interpreted alongside phenotypic susceptibility data. Resistance-associated variants reported in *L. donovani* and *L. infantum* (Section 5.2.1), including loss-of-function changes in MT and Ros3 and aneuploidy at chromosome 13, provide candidate markers for surveillance in *Mundinia* species, although their predictive value in *L. martiniquensis* and *L. orientalis* has not been established and requires direct validation. Phenotypic susceptibility testing performed in conjunction with sequencing has revealed reduced amphotericin B sensitivity in relapsed *L. martiniquensis* isolates [37], demonstrating that integrated phenotype-genotype surveillance is technically feasible in regional reference laboratories. Whether the same framework can detect early shifts in miltefosine susceptibility before they translate into clinical failure depends on the prior establishment of *Mundinia*-specific susceptibility baselines and on systematic phenotypic testing of clinical isolates over time, neither of which is currently in place.

The integration of parasitomic platforms with clinical surveillance is most useful for characterizing baseline parasite populations, identifying species-level shifts in incident cases, and detecting genomic features that warrant phenotypic follow-up. Direct detection of clinically meaningful resistance in patients before treatment failure occurs remains a longer-term aspiration that depends on validated species-specific molecular markers, regional reference panels of phenotypically characterized isolates, and surveillance capacity sustained across the human, animal, and vector interfaces addressed in Section 7.

## 7. Strategic implementation: A One Health framework for miltefosine stewardship in Southeast Asia

The therapeutic case for miltefosine in Southeast Asia rests not only on the available clinical and mechanistic evidence reviewed in Sections 4 and 5 but on the conditions under which the drug would be deployed. The Indian subcontinent experience shows that pharmacokinetic vulnerability, adherence barriers, and inadequately monitored selective pressure must be anticipated before wider deployment [9,88]. For *Mundinia* infections in Thailand and the broader region, where parasite populations remain largely naïve to alkylphospholipid exposure and where epidemiological transmission cycles are incompletely characterized, evidence-based introduction must be framed within a coordinated implementation strategy that integrates clinical use, surveillance, and ecological context.

### 7.1 Principles for evidence-based introduction

Stewardship principles for miltefosine introduction should align with the resistance-aware framing established in Section 5. Combination therapy with liposomal amphotericin B substantially improves cure rates in HIV-coinfected VL patients and reduces relapse, providing the strongest empirical support for combination-based regimens in immunocompromised cohorts [73,83]. The Thai compassionate-use case (Section 4.2) demonstrated that combination induction with miltefosine and liposomal amphotericin B can achieve sustained remission of refractory *L. martiniquensis* infection, where amphotericin B-based regimens alone had failed, while miltefosine monotherapy was insufficient to maintain remission in the context of advanced immunosuppression. This pattern supports combination-based induction as the operational framework for the use of miltefosine in immunocompromised patients with *Mundinia* infections in the region.

Adherence support is equally important given miltefosine’s pharmacokinetic profile, i.e., the terminal elimination half-life of approximately 150–200 hours produces prolonged subtherapeutic exposure when treatment courses are incomplete, generating selection pressure favorable to the emergence of reduced susceptibility [87,88]. Programmatic safeguards used elsewhere in the Indian subcontinent, including supervised drug distribution [11], pregnancy testing and contraception counseling because of teratogenic risk, and structured adherence monitoring across the 28-day oral regimen, are directly transferable to Thai clinical practice.

Pre-deployment isolate monitoring should therefore include the amphotericin B-associated cross-resistance concern described in Section 5.3.

### 7.2 Integrated surveillance across human, animal, and vector interfaces

Thailand already has precedents for multisectoral infectious disease control that could support leishmaniasis stewardship. The Lawa model for opisthorchiasis reduced human infection prevalence from approximately 60% to below 5% through coordinated interventions spanning human health, animal reservoirs, environmental management, and sustained community engagement [190]. Multisectoral surveillance principles have also been applied in Thailand for avian influenza, linking human, animal, and environmental monitoring systems to support early detection and response [191]. These examples indicate that One Health surveillance frameworks are operationally feasible in the Thai public health context, although leishmaniasis implementation remains constrained by fragmented data systems, limited veterinary and entomological capacity, and legal or administrative barriers to data sharing across sectors [191,192].

For *Mundinia* leishmaniasis, this model would require embedding the diagnostic and susceptibility-surveillance workflow described in Section 6 within entomological monitoring and targeted reservoir investigation. The transmission ecology of *L. martiniquensis* and *L. orientalis* in Thailand remains incompletely characterized, with vector competence, reservoir hosts, land-use change, and human mobility remaining active areas of investigation. A One Health stewardship system should therefore prioritize shared reporting structures across clinical, laboratory, veterinary, and entomological sectors rather than treating these activities as separate programs.

### 7.3 Regional implications beyond Thailand

Autochthonous *Mundinia* transmission has been documented across mainland Southeast Asia, making Thailand’s diagnostic, surveillance, and stewardship framework relevant to neighboring settings with similar epidemiological and therapeutic constraints. Regional coordination is important because parasite movement, shared vector ecologies, and treatment-policy differences may allow resistance signals detected in one national context to affect neighboring programmes.

Despite frequent reference to Southeast Asia in regional treatment frameworks, the evidence base informing antileishmanial policy derives substantially from clinical and programmatic data generated in the Indian subcontinent, with limited representation of autochthonous *Mundinia* transmission settings in mainland Southeast Asia. Filling this evidence gap requires regional investment in susceptibility surveillance, harmonized diagnostic standards, and shared platforms for genomic monitoring of resistance-associated markers. Coordinated stewardship across countries with shared *Mundinia* transmission cycles would strengthen the regional capacity to detect epidemiological shifts, respond to therapeutic challenges, and preserve the clinical utility of the limited treatment options currently available.

## 8. Conclusions

Miltefosine represents a valuable oral therapeutic addition for emerging *L. martiniquensis* and *L. orientalis* infections in Southeast Asia, where current parenteral therapies remain limited by toxicity, relapse, and access constraints. The Thai compassionate-use case supports the clinical plausibility of combined miltefosine and liposomal amphotericin B for refractory *L. martiniquensis* infection, but it does not establish generalizable efficacy, optimal dosing, or durable monotherapy. Mechanistic evidence from non-*Mundinia* species and cross-resistance observations in *L. martiniquensis* indicate that regional deployment should proceed with species-resolved diagnosis, susceptibility baselines, relapse monitoring, and phenotype-genotype surveillance. Under these conditions, miltefosine can be framed neither as an unqualified solution nor as a drug to avoid, but as a rational therapeutic option whose durability depends on resistance-aware stewardship.

## Supporting information

S1 Table WHO 2022 guidelines for treatment and secondary prophylaxis of visceral leishmaniasis in HIV co-infected patients in East Africa and South-East Asia. (DOCX)

S1 Fig Phosphatidylcholine biosynthesis through the Kennedy and salvage pathways. The Kennedy pathway comprises two parallel branches operating across cytosolic and endoplasmic reticulum (ER)-associated steps: the CDP-choline branch, initiated by cytosolic choline phosphorylation and culminating in phosphatidylcholine (PC) formation via condensation of CDP-choline with diacylglycerol (DAG); and the CDP-ethanolamine branch, which generates phosphatidylethanolamine (PE). PE may undergo stepwise methylation by phosphatidylethanolamine N-methyltransferase activity (PEMT; mediated by sequential methyltransferases in *Leishmania*) to yield PC, with the relative contribution of this route varying among kinetoplastids. The salvage pathway originates at the mitochondrial inner membrane, where phosphatidylserine (PS) is decarboxylated by phosphatidylserine decarboxylase (PSD) to form PE, which is subsequently transported to ER-associated membranes for further modification. Cofactors and reaction byproducts, including ATP, CTP, S-adenosylmethionine (SAM), ADP, pyrophosphate (PPi), and S-adenosylhomocysteine (SAH), are shown to indicate the energetic and methyl-donor requirements of PC biosynthesis. (!) Circled exclamation marks indicate pathway steps or lipid pools reported to be perturbed during miltefosine exposure, including choline availability, CTP:phosphocholine cytidylyltransferase activity, PEMT-associated methylation, PC depletion, and altered PE abundance. (*) Circled asterisks indicate resistance-associated metabolic features reported in reduced-susceptibility parasites, including altered phosphocholine or methyl-donor metabolism and remodeled baseline PC and PE pools. This schematic is intended as generalized biochemical context and does not imply that all steps are equally active across parasite stages, species, or resistant backgrounds. Created in BioRender. Lerona, P.G. (2026) https://BioRender.com/cv5zdyq.
(TIFF)

S2 Fig Sterol biosynthesis and compositional remodeling through the mevalonate pathway. Acetyl-CoA is condensed to 3-hydroxy-3-methylglutaryl-CoA (HMG-CoA), reduced to mevalonate, and phosphorylated to isopentenyl pyrophosphate (IPP) and dimethylallyl pyrophosphate (DMAPP). These intermediates are sequentially condensed to form geranyl pyrophosphate (GPP), farnesyl pyrophosphate (FPP), and squalene, which is oxidized to 2,3-oxidosqualene and cyclized to lanosterol. In the endoplasmic reticulum, successive demethylation, methylation, isomerization, and desaturation reactions generate ergosterol and related C24-alkylated sterols. Key enzymes, including sterol 14α-demethylase (CYP51), sterol C24-methyltransferase (SMT), and Δ5-sterol desaturase, are shown for pathway context. Miltefosine does not have a functionally validated direct target within the sterol biosynthesis pathway, but drug exposure is associated with acute disruption of sterol homeostasis, whereas resistant parasites exhibit adaptive remodeling of sterol composition. Circled exclamation marks indicate nodes or steps reported to be perturbed during miltefosine exposure. Circled asterisks indicate resistance-associated alterations reported in reduced-susceptibility parasites. These annotations reflect predominantly omics-based and compositional evidence from non-*Mundinia* species and should not be interpreted as direct proof of enzyme inhibition. Created in BioRender. Lerona, P.G. (2026) https://BioRender.com/3xds2ca.
(TIFF)
