# Supplementary material for: Miltefosine and emerging Leishmania (Mundinia) in Southeast Asia: Molecular insights, therapeutic challenges, and future strategic implementation
Source: PLoS Negl Trop Dis. 2026 Jul 31;20(7):e0014555. doi: 10.1371/journal.pntd.0014555 (PMC13426983; doi:10.1371/journal.pntd.0014555)
Supplement: S1 Table — (DOCX) [file pntd.0014555.s001.docx]

# Table S1. WHO 2022 guidelines for treatment and secondary prophylaxis of visceral leishmaniasis in HIV co-infected patients in East Africa and South-East Asia.

| **Acute treatment of VL in HIV co-infected patients** | | | | |
| --- | --- | --- | --- | --- |
| **Region** | **First-choice regimen** | **Alternative if miltefosine unavailable** | **Strength/certainty** |  |
| East Africa | L-AMB up to 30 mg/kg total (5 mg/kg on days 1, 3, 5, 7, 9, 11) + miltefosine 100 mg/day × 28 days | L-AMB up to 40 mg/kg total (5 mg/kg on days 1–5, 10, 17, 24) | Conditional; very-low certainty |  |
| South-East Asia | L-AMB up to 30 mg/kg total (5 mg/kg on days 1, 3, 5, 7, 9, 11) + miltefosine 100 mg/day × 14 days | L-AMB up to 40 mg/kg total (5 mg/kg on days 1–4, 8, 10, 17, 24) | Conditional; very-low certainty |  |

| **Secondary prophylaxis after recovery from the first VL episode** | | | | |
| --- | --- | --- | --- | --- |
| Region | Recommended regimen(s) | Prophylaxis priority | When to stop |  |
| East Africa | Pentamidine isethionate 4 mg/kg (≈300 mg adult) every 3–4 weeks | High-risk patients: not on ART; CD4 < 200; multiple prior VL episodes; failure to achieve clinical/parasitological cure; no CD4 rise | CD4 count ≥ 350 cells/mm³ or undetectable HIV viral load ≥ 6 months and no relapse |  |
| South-East Asia | Amphotericin B deoxycholate 1 mg/kg every 3–4 weeks OR L-AMB 3–5 mg/kg/day every 3–4 weeks | Same high-risk criteria | Same stopping criteria |  |

L-AMB = liposomal amphotericin B; ART = antiretroviral therapy; CD4 count = cluster of differentiation 4 T lymphocyte count.

Reference:

World Health Organization (WHO), 2022. Guideline for the treatment of visceral leishmaniasis in HIV co-infected patients in East Africa and South-East Asia. World Health Organization, Geneva.
